# Gut-kidney axis in IgA nephropathy: Role on mesangial cell metabolism and inflammation

**DOI:** 10.3389/fcell.2022.993716

**Published:** 2022-11-17

**Authors:** Mateus Justi Luvizotto, Luísa Menezes-Silva, Viktoria Woronik, Renato C. Monteiro, Niels Olsen Saraiva Câmara

**Affiliations:** ^1^ Department of Nephrology, Faculty of Medicine, University of Sao Paulo, São Paulo, Brazil; ^2^ Laboratory of Transplantation Immunobiology, Institute of Biomedical Sciences, University of Sao Paulo, São Paulo, Brazil; ^3^ Centre de Recherche sur l’Inflammation, INSERM and CNRS, Université Paris Cité, Paris, France

**Keywords:** IgA nephropathy, kidney, mesangial cells, gut-kidney axis, microbiota

## Abstract

IgA Nephropathy (IgAN) is the commonest primary glomerular disease around the world and represents a significant cause of end-stage renal disease. IgAN is characterized by mesangial deposition of IgA-immune complexes and mesangial expansion. The pathophysiological process includes an abnormally glycosylated IgA1, which is an antigenic target. Autoantibodies specifically recognize galactose-deficient IgA1 forming immune complexes that are amplified in size by the soluble IgA Fc receptor CD89 leading to deposition in the mesangium through interaction with non-classical IgA receptors. The local production of cytokines promotes local inflammation and complement system activation, besides the stimulation of mesangial proliferation. The spectrum of clinical manifestations is quite variable from asymptomatic microscopic hematuria to rapidly progressive glomerulonephritis. Despite all the advances, the pathophysiology of the disease is still not fully elucidated. The mucosal immune system is quoted to be a factor in triggering IgAN and a “gut-kidney axis” is proposed in its development. Furthermore, many recent studies have demonstrated that food intake interferes directly with disease prognosis. In this review, we will discuss how mucosal immunity, microbiota, and nutritional status could be interfering directly with the activation of intrinsic pathways of the mesangial cells, directly resulting in changes in their function, inflammation and development of IgAN.

## 1 Introduction

IgA Nephropathy (IgAN) is the commonest primary glomerular disease around the world and represents an important cause of end-stage renal disease (Wyatt and Julian, 2013). The history of the disease begins in 1968, with the histological description by Jean Berger and Nicole Hinglais (Berger and Hinglais, 1968). Two major hallmarks characterizing IgAN are the deposit of IgA immune complexes followed by consequently mesangial expansion (Berger, 1969; Berger et al., 1975). Before the ‘70s, only IgG was considered a nephritogenic immunoglobulin. After its discovery, many groups including Berger’s group have proposed that alterations in IgA structure resulting in large molecular size with anionic electric charge could be involved in IgAN pathogenesis (Monteiro et al., 1985; Harada et al., 1989). However, only in the ‘90s, this problem started to become unveiled. Based on the previous examination of reduced IgA1 reactivity with jacalin, which is a lectin related to Galactose/N-acetylgalactosamine disaccharide irrespective of sialylation (Andre et al., 1990), a group proposed that aberrancies in the O-glycans formation on the hinge region of IgA1 could be the etiopathogenic factor in IgAN (Mestecky et al., 1993).

The pathophysiological process of IgAN includes an IgA1 molecule lacking O-linked glycosylation in the hinge region, known as galactose-deficient IgA1 (Gd-IgA1) (Tomana et al., 1999). Autoantibodies specifically recognize Gd-IgA1 forming immune complexes with a soluble form of the myeloid IgA Fc receptor, CD89, causing deposits in the mesangial area through interaction with non-classical IgA receptors (Launay et al., 2000; Moura et al., 2008; Suzuki et al., 2009). The local production of cytokines promotes inflammation and complement system activation, besides stimulation of mesangial proliferation (Suzuki H. et al., 2011). Recently, it has been shown that the role of soluble CD89 in IgAN is not only to amplify IgA immune-complex formation but also to directly mediate mesangial proliferation through interaction with CD71 mesangial IgA1 receptor (Cambier et al., 2022). A multi-hit pathogenic hypothesis of IgAN has been suggested to explain the IgAN onset, with 4 factors that must be present during the disease: excessive Gd-IgA1 production, anti-glycan antibodies generation, binding of anti-glycan antibodies to Gd-IgA1 with deposition in the glomerular mesangium, and the local production of cytokines (Suzuki H. et al., 2011).

The spectrum of IgAN clinical manifestations is quite variable ranging from asymptomatic microscopic hematuria to rapidly progressive glomerulonephritis, and the prevalence of the disease varies according to the geographic region and the biopsy policies (Rodrigues et al., 2017). Supportive care is provided in most cases and the recurring treatment is the use of corticosteroids although the protocol is not well established due to its controversial results and applicability (Floege, 2015; Rauen et al., 2020). Despite all the advances, the pathophysiology of the disease is still not fully elucidated. Once the mucosal sites are the major sources of secretory IgA, the mucosal immune system is intriguingly quoted to be a factor in triggering IgAN and a “gut-kidney axis” is thus proposed in its development (Coppo, 2015).

## 2 IgA variations and antigenic responses in IgAN

Human IgA comprehends two isotypes: IgA1, representing 90% of IgA in serum, and IgA2. The main difference between them is the size of their hinge region since IgA2 is shorter and has a sequence of 13 amino acids less when compared to IgA1 (Kerr, 1990). Secretory IgAs (SIgAs) are majorly produced by the plasma cells of the mucosa-associated lymphoid tissues (MALTs) and can exist as monomers or polymers (Woof and Russell, 2011). There is a difference in the production of IgA isotypes, according to the mucosal site. Typically, IgA1 represents the higher fraction of total IgA not only in serum, but also in the nasal, saliva, and men’s genital secretions, while IgA2 represents most of colonic and women’s genital secretions (Pakkanen et al., 2010; Woof and Russell, 2011).

The idea of an altered IgA emerged in the ‘80s, from a study evaluating patients with primary IgAN (Monteiro et al., 1985). When analyzing the eluates from percutaneous kidney biopsies of IgAN patients through high-pressure liquid chromatography, the authors observed that polymeric IgA (pIgA) represented 64% of the total eluted IgA. Moreover, the secretory component binding to pIgA was demonstrated in four out of eight eluates tested, thus showing the polymeric nature of IgA in the mesangial deposition in IgAN. Usually, dimeric IgA is produced majorly at mucosal sites. The polymeric Ig receptor (pIgR), which is expressed by epithelial cells, is responsible for the transport of dimeric IgA into mucosal secretions which is shed to the gut lumen bound to the extracellular part of the pIgR forming the SIgA (Woof and Russell, 2011). Also, the most found isoform in IgAN kidneys is the IgA1. IgA1 can be produced either by bone marrow or at mucosal sites such as the nasopharynx and the intestinal mucosa (Perše and Večerić-Haler, 2019).

Regarding B cell subpopulations during the IgAN development, a clinical cohort study showed that patients exhibited increased tonsillar CD19^+^CD5^+^ B cell levels, which were positively correlated with the histopathological findings in the kidney (Wu et al., 2011). A similar study revealed elevated levels of CD5-expressing B cells in the kidney biopsies of IgAN patients; furthermore, in untreated IgAN patients, these cells secrete great IgA levels and pro-inflammatory cytokines (Yuling et al., 2008). Moreover, B cells may be involved in the synthesis of nephrotoxic IgA independently of T cells, since transfer of murine B cells to subacute combined immunodeficient (SCID) mice, not harboring T cells, was sufficient to cause the establishment of IgA deposits in the kidneys (Suzuki Y. et al., 2011).

Concerning intrinsic characteristics of the mesangial IgA, one of the most consistent abnormalities is the aberrant glycosylation of IgA1 (Smith et al., 2006). The IgA1 heavy chain has a hinge region with six O-glycans, which can carry galactose and sialic acid. This generate different IgA1 O-glycoforms. In IgAN, there is an enhancement in truncated O-glycans recurrence and an increased display of terminal N-acetylgalactosamine (Smith et al., 2006). Hypo-glycosylated IgA1 presents neo-epitopes located in the hinge that become targets of autoantibodies (mainly of IgG isotype). These auto-Abs form immune complexes with Gd-IgA1 and soluble CD89 inducing large aggregates and are prone to mesangial trapping with extracellular matrix components (Monteiro, 2018).

An interesting study using a selective IgA1 protease reverted mesangial deposits and hematuria in a humanized model of IgAN (Lechner et al., 2016). By using a recombinant protease, that cleaves human IgA1 at hinge region, these humanized mice presented a reduction in IgA1-sCD89 complexes. After 1 week of treatment, not only mesangial IgA1 deposits were reduced but also hematuria. Considering the latter evidence, circulating IgA1, more specifically Gd-IgA1, seems directly responsible for IgA1 mesangial deposition and glomerular injury in IgAN at least in mice. However, more studies are necessary to understand the origin of aberrant IgA1 glycosylation and the disease onset.

## 3 Role of gut immune responses, microbiome and dietary intake in the IgAN development: The “gut-kidney axis”

Since the last century, scientific observations have linked IgAN with the mucosal production of IgA (Floege and Feehally, 2016). In 1982, Miura et al. (1982) described that upper respiratory infections induced hematuria in patients with IgAN. Interestingly, in some cases, the hematuria diminished after the administration of antibiotics. Recently, GWAS studies of large cohorts with more than 20,000 patients have identified risk loci for IgAN development (Kiryluk et al., 2014). Among them are genes involved in intestinal mucosal integrity, such as genes related to the synthesis of IgA within the gut and intestinal epithelial barrier integrity, genes related to inflammatory bowel disease (IBD) development, and genes involved in the response to mucosal pathogens (Kiryluk et al., 2014).

The mucosal sites are the major producers of dimeric IgA in the MALTs, and the activation of naïve B cells specific for microbiota antigens at the tertiary follicles drives their differentiation into IgA-producing plasma cells (Cheung and Barratt, 2021). Dendritic and stromal cells at these sites help to induce IgA-class-switching through the production of a proliferation-inducing ligand (April), transforming growth factor beta (TGF-β), interleukin-6 (IL-6) and interleukin-10 (IL-10). IgAN patients have higher levels of circulating IgA, with a significant increase in pIgA. (Coppo, 2018). The findings of pIgA in the renal tissue, mainly in a dimer form (Monteiro et al., 1985), emerges as a possible mucosal origin (Oortwijn et al., 2007). There is not only an elevation of pIgA that may originate from mucosal sites in the circulation of patients with IgAN, but it is also poorly glycosylated and it is found in mesangial IgA deposits (Hiki et al., 2001). Thus, a possible role played by the mucosa in IgAN development favors the existence of a “gut-kidney axis” (Coppo, 2018).

Several evidences support the importance of the relationship between the gut and the kidney in IgAN. First of all, celiac disease and IgAN share molecular mechanisms that involve IgA1, transglutaminase 2 (TG2), and overexpression of Transferrin Receptor (TfR1, or CD71), a non-classical IgA receptor (Abbad et al., 2020). A case study described that a young man, diagnosed with celiac disease, had searched for a clinician prior to treating a kidney disorder. After the diagnosis, the indicated treatment was an ACE inhibitor, oral iron, and a gluten-free diet. The patient achieved nephrotic syndrome remission and showed improvement of both laboratory and clinical parameters related to celiac disease (Habura et al., 2019). Furthermore, a Swedish prospective cohort study found that celiac disease patients are more predisposed to develop IgAN (Welander et al., 2013). Another study investigated 83 kidney biopsies from patients who were identified with inflammatory bowel disease (IBD). IgAN was more frequent than any other glomerular disease, suggesting an association with the IgA1 over-production by the MALTs in these patients (Ambruzs et al., 2014). Besides, patients with both IgAN and IBD comorbidities are more prone to develop end-stage renal disease, according to another Swedish cohort study (Rehnberg et al., 2021).

Several hepato and gastrointestinal inflammatory pathologies including Crohn’s disease, ulcerative colitis, and liver diseases have demonstrated a relation with the onset of IgAN, called secondary IgAN (Szigeti et al., 2007). In addition, in patients with primary IgAN, altered duodenal histopathological findings were observed (Almroth et al., 2006). In these individuals, it was also found a high level of IgA, specific against dietary components, and inflammatory T cells augmentation in the small intestine (Honkanen et al., 2005). Furthermore, an increased number of intraepithelial T lymphocytes in the small intestine and IgA1/IgA2 producer plasma cells were also found in other primary glomerular diseases, suggesting an interruption of oral tolerance in multiple glomerular pathologies that are immune mediated (Rostoker et al., 2001). Some of the findings regarding gut-derived factors and IgAN can be summarized in Table 1, and are better discussed in the following sections.

**TABLE 1:** Intestinal-derived factors contributing to the IgAN pathogenesis.

Influencing factor	Exposure	Intervention/Outcome	References
Microbiota	Antibiotics	1) Broad-spectrum antibiotic treatment abolished Gd-IgA1 deposits and prevented the disease development in young mice	Chemouny et al. (2019)
		2) Rifaximin treatment reduced the deposition of Gd-IgA1 in the glomerulus on an animal model	Di Leo et al. (2021)
	Fecal transplantation	3) Transplant of the microbiota from healthy patients to α1KI-CD89Tg mice reduced albuminuria	Lauriero et al. (2021)
		4) Two patients with IgAN achieved partial clinical remission, after receiving fecal microbiota transplantation	Zhao et al. (2021)
Dietary and nutritional factors	Proteins	1) A ‘low-antigen diet’, with a low content of gluten and proteins, promoted a reduction in immunoglobulins levels, complement fractions’ and fibrinogen deposition in the kidney of IgAN patients	Ferri et al. (1993)
		2) Serum IgA reactivity against gluten antigens is increased in patients with IgAN	Nagy et al. (1988)
		3) Gluten-free mice present a reduction in IgAN-related parameters	Papista et al. (2015)
	Lipids	4) IgAN patients’ lipidome is distinct from healthy controls, and association analysis demonstrated that free fatty acids were positively associated with a high body mass index in IgAN patients	Deng et al. (2021)
	Carbohydrates	5) A significant risk of chronic kidney disease has been associated with a higher carbohydrate daily intake for non-diabetic subjects. More studies in IgAN are necessary	Nam et al. (2019)
	Fibers	6) A recent study investigated the changes in fecal SCFAs in IgAN patients. Levels of propionate, butyrate, acetate, isobutyrate, and hexanoate were reduced in IgAN patients	Chai et al. (2021)

### 3.1 The intestinal microbiota and IgAN development

The intestinal microbiota is essential for body homeostasis, including nutrients absorption, metabolism, and toxin degradation (Chi et al., 2021). Many studies assessed the association between intestinal microbiota and the development of IgAN. A cross-sectional study evaluated the fecal microbiome profile in Chinese patients with IgAN and demonstrated that there is a dysbiosis in gut microbiota, which is associated with IgAN clinical features (Hu et al., 2020). Evidence in recent years indicates differences in fecal and salivary microbiota between individuals with IgAN and healthy controls. The lowest microbial diversity was found in the progressive IgAN patients feces. Levels of fecal and urinary metabolites also differed between patients with and without IgAN and were marked in progressor patients (De Angelis et al., 2014). Another study showed that an augmented microbial load, from different body sites, might be playing a pathogenic role in IgAN (Shah et al., 2021). Thus, an interaction between microbiota and B cells in the MALTs seems to exert an important role in the pathogenesis of IgAN (Bunker et al., 2017).

A recent study compared the gut microbiota, by sequencing 16S rDNA in the feces of patients with IgAN and membranous nephropathy, as well as of healthy controls (Dong et al., 2020). There was a predominance of *Escherichia-Shigella* and *Defluviitaleaceae spp.* in IgAN patients when compared to healthy controls, whereas lower abundances were observed for *Roseburia spp.*, *Lachnospiraceae spp.*, *Clostridium spp.*, and *Fusobacterium spp*. These findings indicate the potential of the microbiota as an IgAN biomarker (Dong et al., 2020). In addition, a group recently developed a transgenic mouse overexpressing BAFF. These mice spontaneously express a hyper-IgA syndrome and an IgAN-like kidney phenotype (McCarthy et al., 2011). When maintained under germ-free conditions, the animals did not develop the kidney phenotype. However, it was re-established when gut microbiota was introduced. Another study performed fecal microbiota transplantation from healthy patients, non-progressors, and progressors IgAN patients to an IgAN humanized mouse model (α1KI-CD89Tg mice), treated with antibiotics prior to the experiments (Lauriero et al., 2021). Microbiota from progressors increased serum BAFF levels and Gd-IgA1, which in turn was associated with increased IgA1 mesangial deposits. Still, transplant of the microbiota from healthy patients to α1KI-CD89Tg mice reduces albuminuria. Furthermore, a case study reported two patients with IgAN who achieved partial clinical remission, after receiving fecal microbiota transplantation (Zhao et al., 2021).

Targeting the microbiota emerges as a potential therapy for IgAN without side effects of more aggressive immunosuppression. In order to modulate the gut microbiota, it has been demonstrated that broad-spectrum antibiotic treatment with amoxicillin, metronidazole, neomycin, and vancomycin of α1KI-CD89Tg mice induced depletion of fecal bacterial load, abolishing Gd-IgA1 deposits and preventing development of the disease in young animals or reversing established disease from 12 week-old mice. Moreover, there was a reduction in proteinuria levels and less glomerular inflammation (Chemouny et al., 2019). Interestingly, there were no alterations in mucosal IgA1^+^ B cells in antibiotic-treated mice, suggesting that microbiota may induce the generation of nephrotoxic Gd-IgA1 by an unknown mechanism rather than altering mucosal IgA1 production. Furthermore, rifaximin, which is a non-absorbable antibiotic, was used to treat α1KI-CD89Tg mice. The treatment reduced the levels of the protein/creatinine ratio in the urine, reduced the serum levels of the Gd-IgA1 complexes with soluble CD89 (sCD89), and reduced the deposition of Gd-IgA1 in the glomerulus (Di Leo et al., 2021). In addition, a phase 2b trial using a new formulation of budesonide (TRF-budesonide) with few systemic effects demonstrated that the modified drug was capable to be delivered to the distal ileum (where most gut MALTs are localized), showing a reduction in proteinuria (Fellström et al., 2017). In conjunction, all these data suggest important participation of the gut microbiota in the production of Gd-IgA1 and in the onset of IgAN. Nevertheless, the causalities between microbiota and kidney diseases are far from being elucidated.

### 3.2 Nutritional status and IgAN: The role of dietary components in IgAN pathogenesis

Reactivity to food antigens is commonly found in IgAN patients. Several studies in the past years had tried to elucidate the relationship between immune responses to dietary antigens and the IgAN development. This association was initially suggested around 40 years ago, when it was suggested that IgAN development was linked to celiac disease and dermatitis herpetiformis (Helin et al., 1983). In the same year, Emancipator et al. experimentally showed that mice orally-immunized with alimentary components had an increased development of IgAN (Emancipator et al., 1983). In addition, an abnormal intestinal permeability can also precedes the IgA responses to some nutrients. In a 5 years follow-up study investigating IgAN patients’ intestinal permeability, high levels of IgA against soy, ovalbumin, gliadin, and oat antigens were detected persistently, towards an abnormal intestinal permeability (Kovács et al., 1996). The intestinal permeability was strongly correlated with the IgA production against soy and other food antigens. In addition, the modulation of intestinal microbiota has been inferred to be correlated with chronic kidney diseases in literature, once dietary changes can disturb the intestinal microbiota composition, which consecutively modulates the intestinal responses, including IgA production (Montemurno et al., 2014; Zoetendal and de Vos, 2014).

#### 3.2.1 Proteins

IgAN patients commonly present reactivity against proteins from their diet (e.g., milk proteins, egg proteins, and gluten). A study described an abnormal immune reactivity in IgAN patients after egg ingestion, which increased the circulating number of immune complexes (Feehally et al., 1987). In addition, another work described that patients with IgAN have increased sensitivity for soy and milk proteins specifically in the rectal mucosal, indicated by the increase of nitric oxide and myeloperoxidase in these regions. Levels of IgG anti-α-lactalbumin, anti- and β-lactoglobulin and casein were also higher in IgAN patients (Kloster Smerud et al., 2010). Since the 1980s, some researches have demonstrated that low-protein diets imposed on animal model and also in patients with chronic kidney disease could preserve glomerular function (Hostetter et al., 1981; Ihle et al., 1989; Klahr et al., 1994).

Contrary to the dietary intake of other macronutrients, proteins also interfere with renal hemodynamics. A high-protein intake increases the intraglomerular pressure, especially through an elevated kidney blood flow. This is necessary to improve the glomerular filtration rate and, thus, the excretion of protein-related metabolites, as nitrogenized products (Ko et al., 2017). Moreover, constantly increased intraglomerular pressure can cause renal damage and loss of kidney function (Kalantar-Zadeh et al., 2020). An observational study in women evaluated the impact of high-protein diets on kidney function and detected a decline in the estimated glomerular filtration rate (GFR) in patients with mild renal insufficiency (Knight et al., 2003). It has also been demonstrated that patients with chronic kidney disease under a low-protein vegetarian diet supplemented with keto analogs were shown to have better-preserved kidney function when compared to patients on the normal diet. Furthermore, it reduces the number of patients in the need of kidney replacement (Garneata et al., 2016). Thus, the nutritional approach to many renal diseases is protein restriction associated with adequate energy supply to prevent malnutrition (Camerotto et al., 2019).

Regarding IgAN, there still is a lack of studies involving other classes of dietary protein antigens rather than gluten antigens. A study evaluated the effect of a “low-antigen-content” diet in patients with active IgAN, which avoided gluten and foods rich in proteins (e.g., eggs and meats), and fed majorly from vegetables, fruits, and rice (Ferri et al., 1993). After the dietetic therapy, they observed a significant reduction in urinary proteins. In addition, kidney biopsies from these IgAN patients showed a reduction of deposits, including immunoglobulins, complement factors and fibrinogen (Ferri et al., 1993).

##### 3.2.1.1 Gluten

Regarding the role of protein antigens in the development of IgAN, gliadin is one of the most studied. In 1988, Nagy et al. (1988) described that serum IgA reactivity against gluten antigens was increased in IgAN patients. Following the first publications, Coppo’s group started a pioneer work that lasts until these days. They started investigating the effects of gluten fractions, notably gliadin, in mice submitted to a gluten-high or gluten-free diet from birth, and observed that mice that received a diet rich in gluten developed more IgA deposits in their kidneys (Coppo et al., 1989). Moreover, the principal gluten lectin fraction, gliadin, predisposed the binding of IgA with mesangial cells *in vitro*. These observations suggested that anti-gliadin antibodies could be involved in the gluten-induced experimental IgAN. A couple of years later, the same research group submitted IgAN patients to a dietary gluten restriction for 6 months (Coppo et al., 1990). The patients had an important reduction in the IgA serum levels and a reduction in the IgA reactivity to gliadin and ovalbumin. Furthermore, a gluten-free diet was correlated with diminished hematuria and proteinuria in these patients but not with renal function. In 1992, Coppo et al. (1992) observed that gliadin could bind to cultured mesangial cells by non-covalent binding, which was reversed by competitive sugars.

More recently, a humanized mouse model, which spontaneously generates IgA mesangial deposits and expresses the human heavy chain of IgA1 and CD89 (the IgA Fc receptor), was studied regarding the pathogenesis of the disease (Berthelot et al., 2012). The humanized mice received a gluten-free diet for three generations. Gluten-free mice presented a reduction in IgAN-related parameters, such as IgA1 mesangial deposition, less inflammation, less deposition of IgA1-sCD89 complexes in the kidney, and lower hematuria. When re-challenged with a diet harboring gluten, these mice recapitulated the symptoms. In addition, it was observed that they presented intestinal symptoms, as well as elevated levels of anti-gliadin IgA1 antibodies. Thus, the development of IgAN may be exacerbated by the interaction between gliadin and sCD89 (Papista et al., 2015). In addition, it was described that in patients with celiac disease, gluten antigens can bind to HLA-DQ2 and DQ8, which can elicit T CD4^+^ cell responses, inducing intestinal epithelial inflammation, thus increasing intestinal permeability to lumen-derived antigens (Tjon et al., 2010).

#### 3.2.2 Lipids

A leading cause of death in patients with chronic kidney disease is cardiovascular disease (Go et al., 2004). These patients have accelerated generation of atherosclerotic plaques due to hyperlipidemia, especially hypertriglyceridemia (Bagdade et al., 1968; Brons et al., 1972; Kaye et al., 1973). Moreover, recent studies showed that when kidney function declines and inflammation rises, the levels of oxidized low-density lipoprotein (LDL) cholesterol increase and dysfunction in high-density lipoprotein cholesterol (HDL) occurs (Bulbul et al., 2018). These evidences support guidelines to recommend the use of lipid-lowering therapy such as statins, in patients with chronic kidney diseases (Dincer et al., 2019).

IgAN, as an important etiology for chronic kidney disease, is also commonly accompanied by dyslipidemia. One recent study aimed to evaluate the lipid species in the plasma of IgAN patients and healthy volunteers. The lipidome of IgAN patients and healthy controls was distinct, and association analysis demonstrated that triacylglycerols containing docosahexaenoic acid, sphingomyelins, and several free fatty acids were positively associated with a high body mass index in IgAN patients (Deng et al., 2021). These findings suggest that lipids can also be targeted as a dietary intervention in the treatment of IgAN.

#### 3.2.3 Carbohydrates

Abnormalities of carbohydrate metabolism have been described in renal diseases since the 1910s (Cramp and Wills, 1975). Seric and tissular high glucose levels generate reactive oxygen species (ROS) as a result of glucose metabolism and auto-oxidation, which generates glycosylated products causing tissue damage (Ha and Lee, 2000). This scenario is seen especially in diabetic nephropathy. A study by Ha *et al.* showed that H_2_O_2_ signaling, mediated by high-glucose levels, increased the fibronectin transcripts and the synthesis of TGF-β1, NF-κB, and activator protein-1 (AP-1) in mesangial cells. These effects were reversible with the administration of antioxidants, suggesting that glucose metabolites can be involved in the activation of mesangial cells (Ha et al., 1997). Another study reported an abnormal response in an intravenous glucose challenge in chronic kidney failure patients; the oral glucose tolerance was also deteriorate in more than half of the uremic patients analyzed (Luke et al., 1964).

Evidence also demonstrates the participation of high-fructose diets in the development of kidney diseases. Experimental data showed that fructose caused hypertrophy of the glomeruli and kidney dysfunction in rats. Harboring-fructose diets promoted an increase in the rats’ kidney weight, such as observed in diabetic rats; however, diabetic rats that received a fructose-low diet did not present the phenotype (Boot-Handford and Heath, 1981). Likewise, it was demonstrated that a high-fructose diet could accelerate chronic disease progression (Gersch et al., 2007). Rats were separated into groups with diets containing high-fructose, high-dextrose, or a normal diet. The high-fructose group presented higher proteinuria and blood urea nitrogen when compared with the chow and dextrose groups. Moreover, they had a decreased creatinine clearance. The high-fructose group also presented larger kidneys, glomerular sclerosis, tubular atrophy and dilatation, and a higher inflammatory cellular infiltration when compared to the other groups. The kidney homogenate from the high-fructose group also showed an increase in the monocyte chemoattractant protein-1 (MCP-1) (Gersch et al., 2007).

Despite these findings, the association of carbohydrate intake with IgAN development is still incipient. In a large-scale prospective cohort study, a significant risk of chronic kidney disease has been associated with a higher carbohydrate daily intake for healthy subjects. On the other hand, there was no significant difference in the incidence of chronic kidney disease risk among those with diabetes mellitus, based on carbohydrate consumption (Nam et al., 2019). Another study used a cohort of elderly adults to find if there are associations between carbohydrate intake and chronic kidney disease, and it reinforced that acute hyperglycemia derived from a diet with lower nutrient intake and more calories could impair kidney function in these subjects (Gopinath et al., 2011).

#### 3.2.4 Fibers

Fiber intake can be beneficial for chronic renal diseases, with activities towards gut microbiota (Camerotto et al., 2019). A study considered the dietary intake of carbohydrates, sugar, starch, cereal, vegetable, and fruit fiber, with the onset of chronic kidney disease. Participants with a higher dietary cereal fiber intake reduced the risk of incident chronic kidney disease by approximately 50% (Nam et al., 2019). A few years later, a cross-sectional study of Swedish elderly men described that a high-fiber diet was associated with improved kidney function and decreased markers of inflammation (Xu H. et al., 2014). Moreover, a high fiber intake was related to survival in kidney dysfunction patients (Xu H. et al., 2014). In addition, high-fiber diets prevented complications related to the development of kidney diseases (Su et al., 2022). Another study evaluated the behavior of dietary fiber intake in relation to mortality in patients with chronic kidney disease in a Korean cohort showing that slight increases in fiber intake significantly reduced the mortality risk in these patients (Kwon et al., 2022).

Short-chain fatty acids (SCFAs), microbiota metabolism-derived products of non-digestible fibers, have been suggested as mediators of the beneficial effects of fibers in preventing kidney diseases. A study with induced type-1 diabetes in mice using streptozotocin showed that a high-fiber intake prevented nephropathy in these animals and, after 12 weeks, they presented less albuminuria, kidney fibrosis and less podocyte injury when compared to diabetic mice under a control diet (Li et al., 2020). In addition, fiber administration modified the microbial profile, increasing the density of *Prevotella* and *Bifidobacterium* genera bacteria, which increased the concentrations of SCFA in both fecal and systemic levels. Furthermore, high-fiber ingestion reduced inflammatory markers, as the expression of genes related to cytokines and fibrosis. Moreover, they induced type-1 diabetes in mice lacking genes related to SCFAs receptors, GPR43 and GPR109A. When treated with SCFAs, diabetic mice were protected from kidney damage, but this did not occur in GPR43 or in GPR109A knockout mice (Li et al., 2020).

A recent study investigated the changes in fecal SCFAs levels in IgAN patients and their correlation with clinical indicators (Chai et al., 2021). Levels of propionate, butyrate, acetate, isobutyrate, and hexanoate were significantly reduced in IgAN patients compared to controls. In addition, the microbiota of IgAN patients was substantially different from the control group. The SCFAs acetate and butyrate were positively associated with *Clostridia*/*Eubacterium* and *Alistipes*. These same groups of bacteria had their relative abundance significantly decreased in IgAN patients. Thus, fiber administration may provide a new therapeutic approach to IgAN patients, especially through the modulation of microbiota.

## 4 Mesangial cells: New clues to understanding the IgAN pathogenesis

### 4.1 The mesangial cell physiology and communication with other kidney cell subsets

The glomerulus is composed of endothelial cells, podocytes, and mesangial cells, each of which having a role in glomerular filtration (Kurihara and Sakai, 2017). In 1933, Zimmerman identified the mesangial cells as the third type of cell in the glomerular tuft (Zimmerman, 1933). However, their functional meaning was elucidated just approximately 50 years after (Sakai and Kriz, 1987). In the 1980s, it was proposed that mesangial cells are special pericytes having contractile properties essential for glomerular filtration (Schlondorff, 1987). Those cells are involved not only in glomerular filtration but also in many functions such as the generation of vasoactive agents, the production of cytokines, and providing structure elements such as collagen. Moreover, mesangial cells can perform endocytosis of macromolecules, including immune complexes (Schlondorff, 1987). In 1998, a group produced monoclonal antibodies that were able to bind to different glomerular cell subsets, and one of them recognized the expression of the Thy1.1 molecule on the surface of mesangial cells in rats (Kurihara et al., 1998). This was confirmed by giving rats a seric injection of the antibody, which caused not only mesangial cell-specific injury but also induced mesangial proliferative glomerulonephritis.

Mesangial cells are located between the capillary loops within the glomeruli. They can both produce and regulate their own extracellular matrix, which is composed mostly of type IV and V collagen, laminin, fibronectin, heparan sulfate, and proteoglycans. During kidney diseases, the matrix composition can be affected (Couchman et al., 1994; Fogo, 1999). Because the mesangial matrix is one that maintains the structural integrity of the glomerular tuft, its regulation is very important and has to be tightly controlled under physiological conditions. The matrix framework maintains the mesangial cells close to the endothelial cells and podocytes, as a single unit (Schlöndorff and Banas, 2009). The principal molecule regulating the matrix turnover by the mesangial cells is TGF-β (Mozes et al., 1999). However, its overproduction is also associated with tissue fibrosis in these sites (Border and Noble, 1997).

Mesangial cells have the capacity in responding to stretch through the generation of soluble factors. Moreover, mesangial cell contractility can be influenced by platelet-derived growth factor (PDGF) (Riser et al., 1996; Gruden et al., 1999; Ingram et al., 1999; Ingram et al., 2000; Riser et al., 2000; Krepinsky et al., 2005). The genetic elimination of PDGF-β causes a failure in the growth of mesangial cells, which makes a single vascular unit without function (Levéen et al., 1994). Due to the cytokine crosstalk among the glomeruli subsets, injury in other glomerular cells such as the podocytes is frequently associated with mesangial cell damage and proliferation (Schlöndorff and Banas, 2009). Mesangial cells can also act as local modulators of innate and adaptive immune responses in the glomeruli. Moreover, mesangial cells and podocytes are able to generate chemokines that have the ability to interact with receptors on their respective cells and exert influence on migration and adherence to the basement membrane (Banas et al., 2002; Ding et al., 2006).

Regarding IgAN, mesangial cells have a central role in disease pathogenesis due to the presence of mesangial IgA deposits. As stated earlier, IgAN is characterized as a mesangial cell proliferative disorder, causing glomerulonephritis. Overexpression of transferrin receptor (CD71) has been identified on mesangial cells of IgAN patients and its high density on the surface these cells allows selective binding of polymeric IgA1 antibodies (Moura et al., 2001). Interestingly, the same receptor is also overexpressed in the apical side of enterocytes of celiac disease patients mediating retrotranscytosis of IgA-gliadin complexes (Matysiak-Budnik et al., 2008). Of note, podocytes do not express receptors for IgA (Lai et al., 2008). The affinity of the mesangial cells for IgA1 causes the accumulation of Gd-IgA1 in the mesangium, which, in turn, stimulates mesangial cell proliferation and increases the production of matrix components. Controversially, the Gd-IgA1 deposition in the mesangial matrix does not necessarily cause kidney disease, as evidenced by *in vitro* experiments with mesangial cells from biopsies of IgAN patients and control samples (Ebefors et al., 2016). The researchers have treated even control-derived and IgAN-patients-derived mesangial cells with Gd-IgA1. However, only IgAN-derived mesangial cells responded to this stimulus by increasing the production of PDGF. In addition, they were more sensitive to recombinant PDGF treatment (Ebefors et al., 2016). Mesangial cells from IgAN patients also produced more IL-6 and had a higher expression of matrix genes compared to control cells. Thus, intrinsic characteristics of mesangial cells could be involved in the onset of the disease, prior to the Gd-IgA1 immunocomplexes deposition in the mesangium. Additionally, targeting PDGF signaling may be a therapeutic alternative to reduce mesangial proliferation in IgAN (Boor et al., 2014). The cytokine expression (e.g., TNF-α, TGF-β1, and MCP-1) was mainly detected in tubulointerstitial space in a study of 96 IgAN patients (Zhang et al., 2021). Furthermore, the cross-talk between mesangial cells and the other glomerular or extra-renal cell types has to be considered (Leung et al., 2018).

Recently, it was described an unexpected role of CD89 in mesangial cells (Cambier et al., 2022). In a study of 67 patients with pediatric IgAN, it was observed that levels of circulating IgA-sCD89 complexes were markedly increased, and the presence of IgA-sCD89 complexes correlated with proteinuria and histological markers of kidney disease. Biopsies from IgAN patients demonstrated the presence of sCD89 in the mesangium. In addition, mesangial cell stimulus *in vitro* with IgA-sCD89 complexes or with recombinant sCD89 induced cell proliferation. They observed that sCD89 is able to bind to CD71, inducing mesangial cell proliferation *via* mTOR pathway. *In vivo*, by giving humanized IgA1 mice an injection of recombinant sCD89, it was observed an induced glomerular proliferation and proteinuria. *In vitro* treatment of human mesangial cells with recombinant CD71 or mTOR inhibitors were able to block sCD89 mediated cell proliferation. This data suggested for the first time the role of sCD89 in IgAN pathogenesis by acting directly in mesangial cells.

### 4.2 Effect of the microbiota and macronutrient intake in the intrinsic pathways of mesangial cells

Dysbiosis and barrier break are not only related to the activation of IgA-producer cells in the gut but also to systemic effects. As previously discussed, alterations of microbiota have been found to be associated with the development of chronic kidney diseases (Ramezani and Raj, 2014). Lipopolysaccharide (LPS), an endotoxin present in gram-negative bacteria, enhances a robust inflammatory response in many cell subtypes. When the gut barrier becomes disrupted, there is an abnormal entry of bacterial contents in the circulation, including LPS. Several studies support the participation of the LPS receptor, toll-like receptor 4 (TLR4), in the pathogenesis of IgAN (Chang and Li, 2020). In 2008, a study demonstrated that the TLR4 activation by bacterial LPS promoted ß-1,3-galactosyl transferase methylation in plasma cells, increasing the seric Gd-IgA1 levels and, thus, IgAN pathogenesis (Qin et al., 2008). Recently, it was described that mononuclear cells in IgAN patients have a higher TLR4 transcripts expression, which correlated with high proteinuria and hematuria in these patients (Coppo et al., 2010). Because bacterial LPS is the main ligand of TLR4, these data further support the gut microbiota role as a promoter of systemic inflammation in IgAN.

Kidney cell subsets can also express TLR4, including mesangial cells. It has been already described that a co-culture of mesangial cells with IgA can be activated through TLR4, which mediates MAPK activation and MCP-1 secretion (Erratum, 2014). MAPK/ERK signaling pathway is related to the secretion of pro-inflammatory cytokines by human mesangial cells. Patients with severe IgAN (considered as disease progressors as displaying proteinuria greater than 1 g/day), showed an increase in MAP/ERK activity in the mesangium, while patients considered as non-progressors did not (Tamouza et al., 2012). The latter study revealed a pathogenic crosstalk between mesangial cells and podocytes. In addition, TLR4 has been associated with the expression of TGF-β1 transcripts during chronic kidney disease (Campbell et al., 2011; Skuginna et al., 2011). Furthermore, TLR4 activation is intrinsically related to the NF-κB translocation to the nucleus, which stimulates the upregulation of pro-inflammatory mediators and contributes to the local immune responses (Chen et al., 2015). TLR4 can also be expressed in other glomerular subsets, such as podocytes, exacerbating local inflammation (Banas et al., 2008). Additionally, an up-regulation of other classes of toll-like receptors (TLRs), such as TLR7, TLR8, and TLR9 was found in IgAN patients (Ciferska et al., 2020). Thus, TLR-activated inflammatory mediators can further trigger tissue damage following direct activation of renal cells (Takeda et al., 2003).

A recent study with human mesangial cells cultured in the presence of IgA showed increased TLR4 expression, as well as production of pro-inflammatory cytokines (e.g., TNF-α, IL-6, MCP-1) and expression of MyD88/NF-κB. The usage of TLR4 shRNA silencing and NF-κB inhibition both reduced the ability of mesangial cells to produce TNF-α, IL-6, and MCP-1. Mesangial cells secreted multiple cytokines when there was increased expression of TLR4, with consequent activation of signaling pathways through the stimulation by SIgA (Zhang et al., 2020). A previous study in lupus nephritis showed that a microRNA, identified as microRNA-16 (miR-16) directly binds to DEC2 on mesangial cells and inactivates the TLR4 signaling. When inhibited, it promotes enhanced proliferation in cultured mesangial cells; however, it is reversed by chloroquine phosphate, a TLR4 antagonist (Qi et al., 2020). In addition, it was demonstrated that a benzylisoquinoline alkaloid, berberine, improved renal function in diabetic rats and mice. A group investigated the effects of berberine on LPS-induced activation of mesangial cells. They showed reduced inflammatory parameters after the treatment of these cells, associated with NF-κB inhibition, which was also associated with suppression of cell proliferation (Jiang et al., 2011).

Aiming to assess whether AMP-activated protein kinase (AMPK) activation would affect the production of inflammatory mediators in mesangial cells, a study evaluated mesangial cells from mice stimulated by LPS/interferon (IFN)-γ (Peairs et al., 2009). Murine mesangial cells cultured in the presence of LPS/IFN-γ showed a decreased threshold to produce inflammatory mediators when treated with an AMPK agonist, 5-amino-4-imidazole carboxamide riboside (AICAR). These observations suggest that induced AMPK activation can be a therapeutic target for mesangial cell inflammation mediated by LPS. As well, the blockade of LPS-mediated signaling in mesangial cells can prevent inflammation. A work demonstrated that the treatment of rat mesangial cells, on a septic acute kidney injury *in vitro* with gold particles under LPS stimulation suppressed pro-inflammatory cytokines (Yuan et al., 2020). Mesangial cell pretreatment with gold clusters was able to significantly reduce LPS-related transcripts, which are also involved in the NF-κB pathway.

Other types of bacterial antigens in mucosal sites can also contribute to mesangial cell activation. *Streptococcus pyogenes* is a highly infectious pathogenic bacterium, often responsible for pharyngitis. A study has shown that the M protein, produced by *S. pyogenes*, is capable to bind to Gd-IgA1 in the serum of IgAN patients, which increases the mesangial deposition of the immunocomplexes, the secretion of IL-6, and C3 activation. Altogether, this scenario promotes mesangial cell proliferation and disease progression (Schmitt et al., 2014). Complement proteins, such as C3, can also activate mesangial cells’ inflammatory pathways. It has been proposed that Gd-IgA1 has a powerful ability to activate the alternative pathway through C3 cleavage (Oortwijn et al., 2006; Shinohara et al., 2006). Thus, the co-deposition of C3 with Gd-IgA1 in the mesangium correlates with the severity and progression of IgAN.

The mesangium is a compartment in continuity with the capillary lumen. In this context, it becomes vulnerable and exposed to abnormal serum molecules (Schlöndorff and Banas, 2009). Therefore, mesangial cells are also sensitive to the serum variations of nutritional factors. The effects of glucose concentrations on the expression of the facilitating glucose transporter (GLUT) were evaluated in mesangial cells from rats. There was an increase in GLUT1 mRNA and GLUT1 protein expression compared to cells chronically adapted to physiological glucose concentrations (Heilig et al., 1997).

The pathophysiological changes of high glucose levels in mesangium may be related to lipid signaling (Boi et al., 2022). Inflammasome-driven IL-1β secretion was initiated by stimulation of human mesangial cells with high glucose. As a result, there was stimulation of PDGF release, which increased IL-1β secretion. IL-1β and PDGF caused a decrease in sphingolipids and the production of phosphorylated sphingoid bases. Furthermore, the blockade of phospholipase cPLA2 release of arachidonic acid diminished mesangial cell proliferation and prostaglandin secretion. Consequently, hyperglycemia is involved in inflammatory and proliferative stimuli by changes in lipid metabolism. (Boi et al., 2022).

Another work evaluated that high glucose and LPS signaling could alter inflammasome in mesangial cells. It was demonstrated that LPS and high glucose increased the levels of nucleotide-binding domain and leucine-rich repeat-containing family, pyrin domain-containing-3 (NLRP3), procaspase-1, and IL-β in mesangial cells. Moreover, N-acetyl-L-cysteine was able to blockade high glucose and LPS signaling (Feng et al., 2016). On the other hand, another study showed that treatment of mesangial cells with antioxidant compounds such as resveratrol attenuated high-glucose-induced effects *in vitro*, reducing cell activation and proliferation. (Xu F. et al., 2014). This was also reproduced *in vivo*, once diabetic mice treated with resveratrol presented reduced numbers of proliferative cells in the glomerulus.

Despite the glomerular capillary network, greater macromolecules can reach the subendothelial and mesangial space, like those coming from the gastrointestinal tract through the food intake. For example, mesangial cells have specific receptors for LDL and LDL in the oxidized form. In addition, they are able to perform micro and macropinocytosis independently of triglyceride receptors (Wanner et al., 1997). Still regarding antigens from food, in 1992, Coppo et al. observed that gliadin could bind to cultured mesangial cells by a non-covalent bond, which was reversed by competitive sugars (Emancipator et al., 1992). To investigate intrinsic activator factors of IgAN mesangial cells, apart from IgA complexes, a study treated human mesangial cells from patients with and without IgAN with Gd-IgA1. More PDGF was expressed and released in mesangial cells of IgAN patients compared with controls. Furthermore, there was an increase in the cells proliferation rate from IgAN patients due to more responsiveness to treatment with PDGF (Ebefors et al., 2016). This led to conclude that intrinsic characteristics of mesangial cells derived from IgAN patients might be important for the development of IgAN.

Furthermore, activated immune cells and their products are associated with a systemic inflammatory response in many kidney diseases. For this to occur, immune cells activated in the periphery (including in the gut) must migrate from the bloodstream to enter the mesangial space. During the cell entrance, this process usually causes damage, culminating with extracellular matrix degradation through the activation of proteinases (Min et al., 2009). Not only is the cell infiltration in the kidney prejudicial, but also cell-derived products. A study investigated the expression of TNF, IL-6, and matrix metalloproteinase 9 in the glomeruli of rodents in a model of diabetic nephropathy (Min et al., 2009). They observed that diabetic rats presented higher levels of glomerular TNF and IL-6, and the macrophage infiltrates co-localized with matrix metalloproteinase 9. In addition, a high-glucose media increased the production of pro-inflammatory cytokines in a mesangial cell-conditioned medium. Besides stimulating the recruitment of human monocytes, TNF and IL-6 also increased the matrix metalloproteinase 9 expression in mesangial cells. Thus, pro-inflammatory cytokines can stimulate mesangial cells to produce factors related to mesangial matrix degradation, which in turn aggravates nephropathies.

Many molecules have been related to the activation of inflammatory pathways during cytokine signaling. However, the Janus kinases (JAK)/STAT may be one of the most studied regarding the recognition of pro-inflammatory factors by many cellular subtypes. The JAK/STAT signaling is a transduction pathway that is universally expressed and involved in diverse biological processes (Banerjee et al., 2017; Xin et al., 2020). These molecules were characterized in glomerular cells for the first time in 1995. JAK1, JAK2, and JAK3 were identified from a total of 24 proteins (Takahashi et al., 1995). Moreover, JAK3 was localized, selectively, in the glomerular epithelia of IgAN patients (Takahashi et al., 1996). A group showed that STAT3 and JAK2 signals were related to the senescence of human glomerular mesangial cells mediated by angiotensin II, promoting autophagy and oxidative stress responses (Zhou et al., 2010; Jiao et al., 2012). The same group evaluated the usage of N-acetylcysteine ​​as an inhibitor of oxidative stress and also of rapamycin (mTOR inhibitor), with consequent suppression of autophagy. The regulation of STAT3/mTOR contributed to the attenuation of the aging process and the antioxidant effects had an impact on autophagy in human mesangial cells (Yang et al., 2019).

**FIGURE 1:**
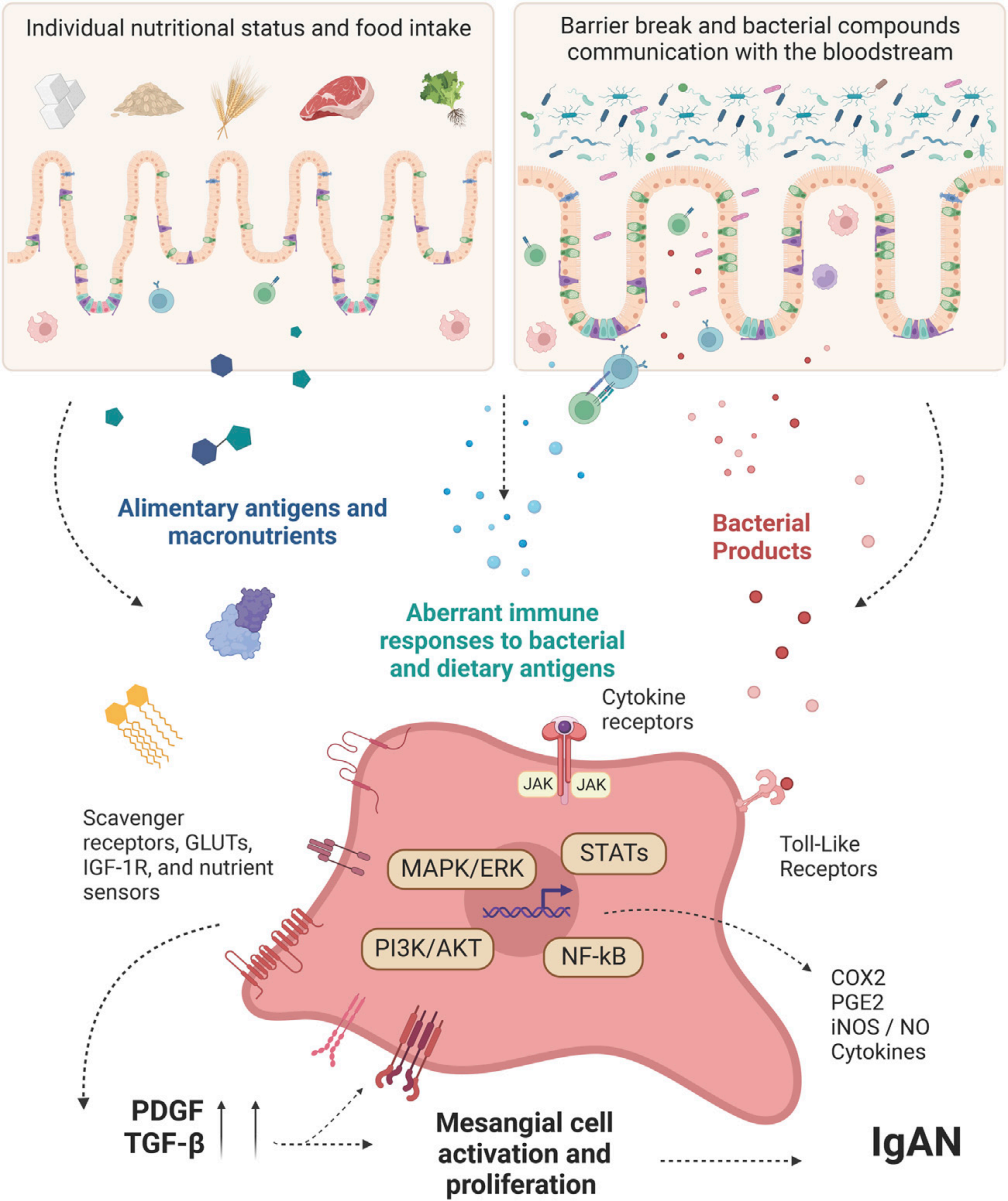
Intestinal-derived factors interfering with mesangial cell-intrinsic pathways activation. Diet-derived molecules and bacterial products from the gut of IgAN patients work as antigens, promoting immune cell activation. After a gut-barrier break, these molecules reach the bloodstream and can be recognized directly by the mesangial cells through specific receptors. Other important factors involved in the activation of mesangial cells are cytokines, which are produced during mucosal activation and can act systemically, reaching the mesangial cells at the glomeruli. At the cellular level, the ligands can promote not only a metabolic shift but also increase the responsiveness regarding inflammatory products by the mesangial cells. This, besides the Gd-IgA1 established model for IgAN, can precede the unique mesangial cell features shown by many IgAN patients, justifying the onset of the disease earlier than the IgA complexes deposition. PDGF: Platelet-derived growth factor; GLUTs: Glucose transporters; NF-κB: Nuclear factor-κB; STATs: Signal transducer and activator of transcription; MAPK: Mitogen-activated protein kinase; ERK: Extracellular signal-regulated kinase; PI3K: phosphoinositide 3-kinase; AKT: protein kinase B; JAK: Janus kinase; COX2: Cyclooxygenase-2; PGE2: Prostaglandin E2; iNOS: Inducible nitric oxide synthase; NO: Nitric oxide; IGF-1R: Insulin-like growth factor 1 receptor; TGF-β: Transforming growth factor beta; IgAN: IgA Nephropathy. Created with BioRender.com.

Unrestricted activation of JAK/STAT pathways contributes to several inflammatory diseases and proliferative disorders and can be involved in mesangial cell activation during IgAN. Recent studies have explored the abnormal activation of these tyrosine kinase receptors in the pathogenesis of IgAN (McAdoo and Tam, 2018). In 2020, Tao et al. (2020) compared patients with histological IgAN with healthy subjects and found that JAK signal transduction was further activated in patients with IgAN compared to controls. It was also observed an increase in pSTAT1 and pSTAT3 activities in glomerular and tubulointerstitial areas of the kidney in IgAN patients. Similarly, cytokines related to the JAK/STAT pathway activation are also increased during IgAN. Not only is the intrarenal production of cytokines augmented (Lim et al., 2003), but also systemically. A study demonstrated a significant difference between nine serum cytokine signatures of IgAN and healthy controls, such as IL-5, IL-7, IL-15, VEGF, IL-10, IL-12p40, IL-1RA, MCP-3, and MIP-1B (Woo et al., 2012). There were also altered serum levels of IFN-γ and TNF-α in these patients (Rostoker et al., 1998). Since IgAN are usually accompanied by gut inflammatory responses (as discussed in the above sections), targeting these inflammatory signaling pathways in mesangial cells, including JAK/STAT, may help us to improve treatment of IgAN (Chang and Li, 2020). All the appointments and pathways related to the mesangial cells’ activation are summarized in Figure 1.

## 5 Conclusions and future perspectives

This review summarizes the interaction between mucosal factors, such as microbiota, macronutrients, mucosal immunity and pro-inflammatory factors, and the putative intrinsic inflammatory pathways that could be activated in mesangial cells during IgAN. Once IgAN is the prototype of mesangioproliferative glomerulonephritis, mesangial cells are a central part of the disease pathophysiology and modulate the inflammatory response through different pathways. Moreover, environmental factors such as dietary intake and microbiota dysbiosis can trigger the disease through direct mesangial cell activation.

There is still no specific treatment for IgAN. The current approach consists of treating risk factors such as hypertension, dyslipidemia, and the renin-angiotensin-aldosterone system blockade. If proteinuria persists greater than 1 g per day, corticosteroids are considered, but with inconsistent results (Group KDIGOKGDW, 2021). Considering the gut-kidney axis and its actions directly on mesangial cells, the blockade of TLR-mediated signaling (and, thus, the inhibition of NF-κB pathway), the usage of AMPK agonists and treatment with antioxidants as resveratrol have demonstrated promisor results in experimental studies and can help to prevent mesangial cells’ inflammation (Peairs et al., 2009; Xu F. et al., 2014; Feng et al., 2016; Yuan et al., 2020). We truly believe that, in the near future, we will be able to use specific pathways as a target to block mesangial cell inflammation as agonists to the treatment, which could be a promising field in the prevention of progression of the IgAN development. Further studies are needed to understand the complexity of molecular interactions between mucosal sites and kidneys to identify new crucial pathways involved in the pathogenesis bringing potential targets for specific treatments in IgAN.
